# Trabecular Bone Score (TBS) in Patients with Early Ankylosing Spondylitis—Limited Utility

**DOI:** 10.3390/jcm10225373

**Published:** 2021-11-18

**Authors:** Anna Nowakowska-Płaza, Jakub Wroński, Iwona Sudoł-Szopińska, Piotr Głuszko

**Affiliations:** 1 Department of Rheumatology, National Institute of Geriatrics, Rheumatology and Rehabilitation, 02-637 Warsaw, Poland; andzior_n@o2.pl (A.N.-P.); piotr.gluszko@spartanska.pl (P.G.); 2 Department of Radiology, National Institute of Geriatrics, Rheumatology and Rehabilitation, 02-637 Warsaw, Poland; iwona.sudol-szopinska@spartanska.pl

**Keywords:** osteoporosis, ankylosing spondylitis, DXA, trabecular bone score

## Abstract

Purpose: Ankylosing spondylitis (AS) not only results in pathological ossification of the spine, but can also be associated with osteoporosis. Due to the presence of syndesmophytes and possible involvement of the hip joints, classical dual X-ray absorptiometry (DXA) is of limited use in patients with advanced stages of AS. Trabecular bone score (TBS) is a method complementary to DXA, providing additional information about bone microarchitecture. There is a growing body of evidence for the usefulness of TBS in AS patients. The aim of this study was to assess the clinical utility of TBS in patients with AS. Methods: Patients with AS underwent DXA with additional TBS assessment. A cross-sectional analysis of the frequency of osteoporosis and bone microarchitecture deterioration and their association with patients’ characteristics was done. Results: A total of 51 male patients, mean age 40.7 years, were enrolled. Osteoporosis was diagnosed in seven patients (13.7%). Lumbar bone mineral density (BMD) was higher (*p* < 0.001) than femoral BMD, indicating abnormal BMD readings in the spine caused by syndesmophytes. Patients with DXA-diagnosed osteoporosis had lower TBS (*p* = 0.03) and TBS T-score (*p* = 0.043) values compared to patients without osteoporosis. However, disturbed bone microarchitecture (TBS < 1.23) was present in only three patients (5.9%). None of the patients had a history of an osteoporotic fracture. A lower TBS T-score (*p* = 0.032) was demonstrated in patients with sacroiliitis grade 4 than in patients with sacroiliitis grade 2, with no significant differences in BMD and T-score values. Conclusion: Among patients with early AS, the clinical utility of TBS is limited—it does not add value to DXA.

## 1. Introduction

Ankylosing spondylitis (AS) is a chronic progressive autoinflammatory disease predominantly affecting the axial skeleton. The progressive ossification of the vertebral column resulting from chronic inflammation leads gradually to irreversible loss of spinal mobility. However, in the first phase of the disease, bone erosion dominates over pathological bone formation. Therefore, inflammatory processes observed in AS patients may also lead to osteoporosis. The incidence of osteoporosis in AS according to literature is estimated to be about two times higher than in the general population [1]. Currently, in AS patients, it is proposed that screening of osteoporosis should be performed after 10 years of disease duration [2], but there is little evidence as to which diagnostic method should be used [3]. Traditional dual X-ray absorptiometry (DXA) of the lumbar spine enables diagnosis only in the early stage of the AS. When syndesmophytes and calcification around the spine appear, lumbar DXA results are artificially high [2,4]. At the later stages of AS, only femoral DXA gives reliable results—provided that the hip joints are not affected. This is why the search for novel diagnostic tools for osteoporosis in AS patients is ongoing.

As the process of osteoporosis in AS results from the loss of the trabecular bone [2,4], using diagnostic tools assessing trabecular bone microarchitecture in AS patients has been studied for several years. Currently, the most widely used tool for the evaluation of bone microarchitecture is the trabecular bone score (TBS). TBS iNsight™ is a software tool available for DXA scanners that enables the assessment of bone texture (an index correlated to bone microarchitecture) based on gray-level variations in DXA scans of the lumbar spine. The result is expressed as TBS, with higher scores in patients with better quality bone microarchitecture. TBS is a method complementary to DXA, providing additional information about the bone structure, and is an independent predictor of fracture risk [5]. European guidance for the diagnosis and management of osteoporosis in postmenopausal women emphasizes that TBS can supplement the Fracture Risk Assessment Tool (FRAX) in the estimation of 10-year risk of fractures [6]. TBS proved to be particularly useful in some types of secondary osteoporosis, where bone quality is especially affected [7]. Several studies have shown the usefulness of TBS in assessing bone microarchitecture in glucocorticoid-induced osteoporosis [8], osteoporosis associated with diabetes mellitus [9], primary hyperparathyroidism [10], and chronic kidney disease [11].

There is a growing body of evidence for the usefulness of TBS in AS patients, including TBS being an independent predictor of fracture risk in AS patients [12,13,14,15]. The aim of this study was to assess the clinical utility of TBS in fracture risk assessment in male patients with AS. To our knowledge, this is the first study performed in the Polish or European population. Thus far, similar studies were conducted only in South Korea, Canada, Brazil, and Egypt.

## 2. Patients and Methods

The cross-sectional study was conducted at the Department of Radiology in the National Institute of Geriatrics, Rheumatology, and Rehabilitation in Warsaw, Poland. Patients with ankylosing spondylitis according to modified New York criteria were enrolled. The exclusion criteria were: age < 20 years, body mass index (BMI) < 17 kg/m^2^ or >37 kg/m^2^ (criteria provided by the TBS software manufacturer [16]), patients with diabetes mellitus, primary hyperparathyroidism, chronic kidney disease, and patients with significant motor impairment preventing proper DXA examination. All recruited patients underwent DXA with additional TBS assessment. The study protocol was approved by the hospital bioethics committee (KBT-2/3/2019). All participants signed informed consent for inclusion in the study. The study was conducted according to the Declaration of Helsinki.

DXA scans were performed using a Hologic Discovery A densitometer. DXA reports included the bone mineral density (BMD) value, expressed as grams per square centimeter (g/cm^2^), and the T-score and Z-score values. In all analyses of the femur, DXA reports the lower BMD value of the femoral neck, or the total hip was used. As recommended by the International Society for Clinical Densitometry (ISCD), the T-score was taken into account for men over 50 (the study included only male participants), osteoporosis was diagnosed when the T-score was ≤−2.5, and osteopenia when the T-score was <−1 and >−2.5. In men under 50 years, according to ISCD’s official position, Z-score values are the preferred method of BMD reporting, with a Z-score of −2.0 or lower considered as low bone density for chronologic age. Additionally, men at any age with secondary causes of low BMD (e.g., AS) may be diagnosed clinically with osteoporosis supported by findings of low BMD. Therefore, for the purpose of the study, we diagnosed osteoporosis in men under 50 years based on a Z-score ≤ −2.0 [17]. TBS was assessed by automated analysis of lumbar spine DXA results using TBS iNsight™ version 3.0.3.0 software (Medimaps, Geneva, Switzerland). The TBS reports included the absolute TBS value and the TBS T-score for the sum of L1-L4 vertebrae. The absolute values of TBS were divided into those suggesting disturbed bone microarchitecture (<1.23), intermediate (≥1.23 and <1.31), or normal (≥1.31) [18]. For analysis of radiographic advancement of sacroiliac joints, the highest degree of sacroiliitis on either side was noted.

We analyzed the frequency of osteoporosis and disturbed microarchitecture depending on demographic and clinical characteristics of patients, the correlation between TBS and classical DXA parameters, and the correlation of DXA/TBS parameters with patients’ characteristics. The compliance of the data with the normal distribution was assessed using the Kolmogorov–Smirnov test. The significance of the observed differences between the two groups was assessed using the Student’s t-test for variables with a normal distribution, the Mann–Whitney U test for variables without a normal distribution, and for categorical variables, the Chi-square test or the Fisher’s exact test (for tables with values less than 5). For more than two groups with normal distribution, we used analysis of variance with post hoc analysis with the Bonferroni test. For more than two groups without normal distribution, we used the Kruskal–Wallis test and Dunn’s test, respectively. The correlation was assessed using the Pearson correlation coefficient for parametric variables and Spearman’s rank correlation coefficient with non-parametric variables. The significance of the correlation after adjusting for the confounding factors was checked by linear regression. The multivariate ANCOVA analysis was performed to identify the predictors of reduced density and bone architectural disturbances. Statistical significance was set at *p* < 0.05. Statistical analysis was performed using Statistica 13.1 software (StatSoft Polska, Kraków, Poland).

## 3. Results

A total of 51 patients (all male) were enrolled. Patients were relatively young (mean 41 years), with a short duration of the disease (median 10 years from symptoms onset), and only 37.3% had the most advanced radiographical changes (sacroiliitis grade 4). The patients’ full characteristics are presented in Table 1.

Osteoporosis was diagnosed in 7 patients (13.7%), in all based on lumbar DXA (2 had additionally osteoporosis in femoral DXA). Disturbed bone microarchitecture (TBS < 1.23) was present in 3 patients (5.9%), of which 2 also had osteoporosis based on DXA. The patients’ full bone status is presented in Table 2. None of the patients had a history of an osteoporotic fracture.

**Figure 1:**
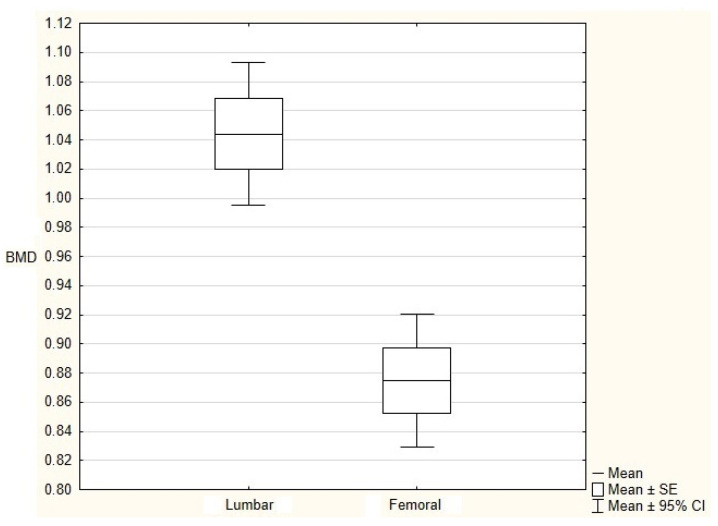
Femoral and lumbar bone mineral density (BMD).

**Figure 2:**
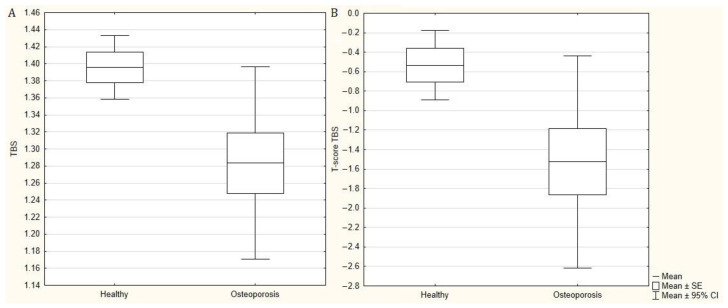
(**A**) Trabecular bone score (TBS) and (**B**) trabecular bone score T-score (T-score TBS) in patients with normal bone mineral density and with osteoporosis.

Lumbar BMD was significantly higher (*p* < 0.001) than femoral BMD (Figure 1). Although there was no correlation between TBS and BMD results, regardless of the location used, patients with DXA-diagnosed osteoporosis had significantly lower TBS (*p* = 0.03) and TBS T-score (*p* = 0.043) values compared to patients without osteoporosis (Figure 2). In the comparative analysis of patients divided into groups depending on the advancement of radiographic changes in the sacroiliac joints, a significantly (*p* = 0.032) lower TBS T-score and borderline (*p* = 0.052) lower TBS were demonstrated in patients with sacroiliitis grade 4 than in patients with sacroiliitis grade 2, with no significant differences in BMD and T-score values.

Among the assessed risk factors for the occurrence of BMD and bone microarchitectural disorders, the univariate analysis showed significantly lower values of TBS (*p* = 0.047) and TBS T-score (*p* = 0.037) in biologically treated patients compared to patients not treated biologically (Table 2). However, these associations were not confirmed in the multivariate analysis. After adjusting for cofounding factors, only the positive correlation of DXA results with the patients’ BMI remained significant.

## 4. Discussion

Osteoporosis in AS patients is mediated by pro-inflammatory cytokines, such as TNF alpha, Il-17, Il-1, which activate osteoclastogenesis by overexpressing the RANKL [2,19]. In addition, significantly lower concentrations of osteoprotegerin, RANKL neutralizing protein, are observed in AS patients, which results in osteoclast differentiation and destruction of bone tissue [20]. The prevalence of osteoporosis in AS according to literature is estimated to be between 13 and 25% [2,4,21] and increases with patients’ age and duration of the disease. The systematic review performed by Weijden et al. showed the prevalence of osteoporosis to be 13% in AS patients with disease duration shorter than 10 years [21]—in our patients with a similar duration of the disease (mean 12.9 years) the prevalence was 13.7%. Despite the short duration of the disease, the mean value of lumbar BMD was significantly higher than the value of femoral BMD, indicating overestimation of BMD in the spine due to syndesmophytes.

Although none of the patients in our study had a history of fractures, studies in AS patients suggest that fractures occur in 10–12% of patients but are associated with advanced age and longstanding disease [2,22,23]. The diagnosis may be problematic because it most often concerns vertebral fractures, mainly at the thoracic spine. Chronic back pain, which is characteristic of AS, may mask the pain associated with the occurrence of a vertebral fracture, and severe thoracic kyphosis is attributed to the typical course of the disease.

TBS in AS patients was assessed in several studies. Reported ranges of low TBS (<1.31) in AS patients ranged between 7 and 87.5% [13,14,15,24,25,26], with disturbed microarchitecture (TBS < 1.23) reported as between 9 and 47.5% of AS patients [13,14,24,25] and partially disturbed microarchitecture (TBS ≥ 1.23 and <1.31) as between 10 and 40% of AS patients [13,14,24,25]. This large discrepancy is due to the wide variety of patients included in the studies conducted so far. The effect of age, disease duration, radiological advancement, and disease activity on bone architecture disorders has been found in several studies. Patients with less advanced radiological changes assessed by mSASSS had better TBS [15,24,26,27,28]; therefore, better TBS results were shown by younger patients [14,26] and patients with shorter duration of the disease [28]. Similarly, the influence of high disease activity—as assessed by ASDAS [26], inflammatory markers [24,26,28], and active radiological changes in MR [29]—was proven to result in lower TBS values. Importantly, the relationship between disease activity and TBS value has been demonstrated in a prospective study [26]. The lowest rates of disturbed bone microarchitecture were presented in a series of studies by Kang et al.—which included relatively young patients with a short duration of the disease [13,25,26], with the lowest rates in AS patients in remission [26]. This is consistent with the results of our study.

In our study, only three patients (5.9%) had disturbed bone microarchitecture, of which two also had osteoporosis based on DXA. Partially disturbed bone microarchitecture occurred in an additional seven patients (13.7%). There were also no significant correlations between the values of TBS and TBS T-score with BMD, T-score, and Z-score, regardless of the location. However, in patients with advanced radiographic changes in the sacroiliac joints (grade 4), the TBS and TBS T-scores were significantly lower compared to patients with AS with initial radiographic changes (sacroiliitis grade 2). Such a relationship has not been demonstrated for BMD parameters. This testifies to the progressive disturbance of the bone tissue microarchitecture with the disease progression and is consistent with other publications. In our study, the univariate analysis also showed lower values of TBS and T-score of TBS in biologically treated patients compared to patients not treated biologically, although after taking into account confounding factors, this association was no longer statistically significant.

The clinically most important question is whether TBS in AS patients is useful, providing additional information over classical DXA, especially with regard to the prevention of osteoporotic fractures. There are studies that have shown the utility of TBS in predicting fractures in patients with AS, independent of FRAX [12]. The ability to predict fractures in patients with AS was assessed as comparable to [15] or greater than [13] the predictive ability of BMD. There is also discussion whether low TBS may be a marker of faster radiographic disease progression with conflicting published up to date [25,27]. However, as our TBS study shows, it is not a universal tool for assessing bone microarchitecture in every patient with AS. In our group of patients—relatively young with a short disease duration—TBS showed fewer bone disorders than classic DXA. In our opinion, future research should, therefore, focus on identifying a specific group of patients with AS for whom TBS is worth performing.

Our study presents several limitations. The biggest limitation of our study is the relatively small sample size and the cross-sectional character of the study. Longitudinal observational studies that can demonstrate the usefulness of TBS in predicting osteoporotic fractures in AS patients would have the greatest clinical value. Another limitation of our study is the fact that we assessed only the advancement of radiological changes in the sacroiliac joints, but not in the spine by SASSS/mSASSS. Lastly, our group of patients did not include patients with low-energy fractures; therefore, we could not compare TBS between patients with and without fractures. The greatest strength of our study is the assessment of the usefulness of TBS in a specific group of patients—young male patients with a short duration of AS. In this group, TBS shows no additional benefits over DXA, which should be taken into account in clinical practice.

## Figures and Tables

**Table 1:** Patients’ characteristics. GCs—glucocorticoids, NSAIDs—non-steroid anti-inflammatory drugs.

Sex, number (%)	
Men	51 (100%)
Age, mean (±SD)	40.7 (±11.3)
BMI, mean (±SD)	25.8 (±4.1)
Disease duration since symptoms onset, median (min, max)	10 (2, 40)
HLA B27, number (%)	45 (91.8%)
Sacroiliitis grade, number (%)	
II	15 (29.4%)
III	17 (33.3%)
IV	19 (37.3%)
Biological treatment, number (%)	17 (33.3%)
adalimumab	10 (58.8%)
golimumab	3 (17.6%)
etanercept	2 (11.8%)
infliksymab	1 (5.9%)
sekukinumab	1 (5.9%)
treatment duration in months, mean (±SD)	39.1 (±30.6)
NSAIDs treatment, number (%)	34 (82.9%)
continuous	32 (78%)
max doses	29 (70.7%)
GCs treatment, number (%)	5 (12.2%)
treatment duration in months, mean (±SD)	72.8 (±60.1)
Smoking, number (%)	15 (37.5%)
pack-years, mean (± SD)	16.1 (±11.8)

**Table 2:** The incidence (number, %) of disturbed (<1.23), intermediate (≥1.23 and <1.31), or normal (≥1.31) bone microarchitecture and BMD status—osteoporosis (T-score ≤ 2.5 in patients aged ≥50 or Z-score ≤ −2 in patients aged <50), osteopenia (T-score < −1 and >−2.5), and healthy (T-score was ≥−1 and Z-score > −2 in patients aged <50). DXA—dual X-ray absorptiometry.

	Disturbed Bone Microarchitecture	Intermediate Bone Microarchitecture	Normal Bone Microarchitecture
- Osteoporosis			
lumbar DXA	2 (3.9%)	1 (2%)	4 (7.8%)
femoral DXA	-	1 (2%)	1 (2%)
- Osteopenia			
lumbar DXA	-	2 (3.9)	11 (21.6%)
femoral DXA	1 (2%)	3 (5.9%)	8 (15.7%)
- Healthy			
lumbar DXA	1 (2%)	4 (7.8%)	26 (51%)
femoral DXA	2 (3.9%)	3 (5.9%)	32 (62.7%)

## Data Availability

The data underlying this article will be shared on reasonable request to the corresponding author.
